# Convergent and divergent brain–cognition relationships during development revealed by cross-sectional and longitudinal analyses in the ABCD Study

**DOI:** 10.1101/2025.06.06.658294

**Published:** 2025-06-09

**Authors:** Yapei Xie, Shaoshi Zhang, Csaba Orban, Leon Qi Rong Ooi, Ru Kong, Dorothea L. Floris, Xi-Nian Zuo, Elvisha Dhamala, Avram J Holmes, Lucina Q. Uddin, Thomas E Nichols, Adriana Di Martino, B.T. Thomas Yeo

## Abstract

How brain networks and cognition co-evolve during development remains poorly understood. Using longitudinal data collected at baseline and Year 2 from 2,949 individuals (ages 8.9-13.5) in the Adolescent Brain Cognitive Development (ABCD) Study, we show that baseline resting-state functional connectivity (FC) more strongly predicts future cognitive ability than concurrent cognitive ability. Models trained on baseline FC to predict baseline cognition generalize better to Year 2 data, suggesting that brain–cognition relationships strengthen over time. Intriguingly, baseline FC outperforms longitudinal FC change in predicting future cognitive ability. Differences in measurement reliability do not fully explain this discrepancy: although FC change is less reliable (intraclass correlation, ICC = 0.24) than baseline FC (ICC = 0.56), matching baseline FC’s reliability by shortening scan time only partially narrows the predictive gap. Furthermore, neither baseline FC nor FC change meaningfully predicts longitudinal change in cognitive ability. We also identify converging and diverging predictive network features across cross-sectional and longitudinal models of brain–cognition relationships, revealing a multivariate twist on Simpson’s paradox. Together, these findings suggest that during early adolescence, stable individual differences in brain functional network organization exert a stronger influence on future cognitive outcomes than short-term changes.

## Full Text Availability

The license terms selected by the author(s) for this preprint version do not permit archiving in PMC. The full text is available from the preprint server.

